# Comparison of Two Preparation Methods for Platelet-Rich Plasma Eye Drops for Release of Growth Factors and De-Epithelization Rabbit Model

**DOI:** 10.1155/2020/6634744

**Published:** 2020-12-01

**Authors:** Tatsuhiko Kobayashi, Takashi Suzuki, Tomohiko Saito, Takashi Itokawa, Yuichi Hori

**Affiliations:** ^1^Department of Ophthalmology, Toho University Graduate School of Medicine, Tokyo, Japan; ^2^Department of Ophthalmology, School of Medicine, Toho University, Tokyo, Japan

## Abstract

**Purpose:**

To compare two platelet-rich plasma (PRP) preparation methods (double spin (D-PRP) and TriCell PRP (T-PRP)) for eye drops, concentration yields of platelets and other cells, release of growth factors, and efficacy of the de-epithelization rabbit model.

**Methods:**

PRP was extracted by D-PRP and T-PRP from 30 ml blood from healthy adults. After extraction, platelets and leukocytes were counted. D-PRP and T-PRP were preserved during A: 1 h storage at room temperature, B: 1 h storage at −20°C, C: 24 h storage at 4°C, and D: 24 h storage at −20°C. Platelet-derived growth factor (PDGF) was measured. Freezing/thawing PRP eye drops and control were instilled in the de-epithelization rabbit model four times per day for 5 days. Histology was compared between eyes treated with control, D-PRP, and T-PRP.

**Results:**

14 ml of D-PRP and 4 ml of T-PRP were extracted from 30 ml whole blood samples. D-PRP and T-PRP had 41.36 ± 8.43 × 10^4^ and 67.02 ± 13.55 × 10^4^ platelets and 0.41 ± 0.24 × 10^3^/ml and 10.09 ± 4.29 × 10^3^/ml leucocytes, respectively. In the four storage methods, PDGF concentrations in T-PRP were higher than those in D-PRP eye drops. Freezing/thawing D-PRP and T-PRP increased PDGF concentrations. Histology showed corneal epithelium thickness in T-PRP-treated eyes after healing (38.41 ± 9.10 *μ*m) was significantly higher than that in control-treated (27.77 ± 4.76 *μ*m) and D-PRP-treated eyes (18.32 ± 3.14 *μ*m) (*P* < 0.05). There was no corneal damage with inflammation in corneal stroma and epithelium of all tested groups. Electron microscopy revealed strong adhesion between cell junctions in T-PRP-treated eyes.

**Conclusions:**

Freezing/thawing of PRP extracted with the T-PRP kit may result in high platelet and leukocyte concentration and produce high PDGF concentration. PRP eye drops including leucocytes could increase thickness of corneal epithelium without corneal inflammation.

## 1. Introduction

Ocular surface disorders caused by infection, inflammation, and injury may cause a delay in wound healing of the corneal tissue such as the epithelium and stroma, ultimately resulting in visual disturbance and persistent pain. To activate the rapid regeneration of the corneal tissue, several growth factors such as epithelial growth factor (EGF), nerve growth factor (NGF), and fibroblast growth factor (FGF), as well as components of the extracellular matrix (ECM) such as fibronectin, are used as topical treatment [1–4]. However, because it may be difficult to extract and maintain these components and such process may be expensive, they are not widely used. Instead of pure components, eye drops derived from blood and body derivatives are used due to the stimulation of cellular proliferation and migration via the supply of an active mixture of growth factors and cytokines at the ocular surface. As these derivatives can be obtained from the patient's own peripheral blood, there are few side effects and immune reaction. Many studies have reported that autologous serum eye drops were effective in the treatment of persistent corneal epithelial defect (PED) and dry eye [5–7]. To fabricate autologous serum eye drops, the blood collected from patients is centrifuged and the supernatant is retrieved and used as eye drops. Recent works reported that platelet-rich plasma (PRP) eye drops are effective for the treatment of corneal diseases such as PED and recurrent corneal erosion (RCE) [8–19]. Because platelets release many growth factors such as platelet-derived growth factor (PDGF) and EGF, PRP is expected to promote rapid wound healing of the corneal epithelium and stromal tissue. Although various methods of PRP extraction from blood have been reported, a standardized preparation method for the PRP eye drop has not been determined. Single- or double-spin methods are generally among the extraction methods used [8, 20]. Using spin methods, it is sometimes difficult to collect pure platelets and concentrate platelets from blood. Recently, several kits for PRP extraction have been made commercially available and can be used to easily and reproductively collect high concentrations of platelets. The TriCell PRP Kit (Yamato Scientific Co., Ltd., Tokyo, Japan) is designed to be used for the safe and rapid preparation of an autologous PRP from a sample of blood [21]. Because the kit is a closed system, there may be few contaminations by preparers. Although PRP extracted using kits is widely used in the field of dermatology and other fields, including ophthalmology [22, 23], little is known about its efficacy when extracted using PRP extraction kits in the ophthalmology field.

In the clinical setting, following collection of blood and extraction of PRP, PRP eye drops should be stocked prior to use as treatment. To determine the efficacy of PRP, PRP containing higher growth factors should be used. However, little is known about the best preservative condition for the good efficacy of PRP eye drops.

PRP eye drops could promote epithelial and stromal regeneration via growth factors. However, little is known about the exact mechanism of PRP in the corneal tissue and the corneal components that are stimulated by the PRP eye drop. Thus, it is necessary to recognize the action of PRP in the corneal tissue for its clinical application. We hypothesized PRP extracted using kits would be more effective compared to PRP prepared by spin methods, due to the higher concentration of growth factors.

In this study, we measured and compared the growth factors and efficacy in vitro in PRP eye drops produced using the PRP extraction kit and conventional methods.

## 2. Methods

### 2.1. Blood Sample

Blood samples were collected from healthy volunteers. Informed consent was obtained from patients after the nature and possible consequences of the study were explained. This study was approved by the Ethics Committee of Faculty of Medicine, Toho University (no. A18115) and adhered to the tenets of the Declaration of Helsinki.

### 2.2. PRP Extraction

#### 2.2.1. Double-Spin Methods

PRP extraction was performed using double-spin methods described elsewhere [20]. In brief, 30 ml of whole blood with 3 ml of anticoagulant citrate dextrose solution and solution A (ACD-A) were centrifuged with 200 g for 10 min. The supernatant was aspirated and recentrifuged at 300 g for 5 min. Finally, serum was carefully removed while avoiding aspiration of platelet cells and the remaining serum and platelet cells were mixed.

#### 2.2.2. TriCell PRP Extraction Kit

**Figure 1:**
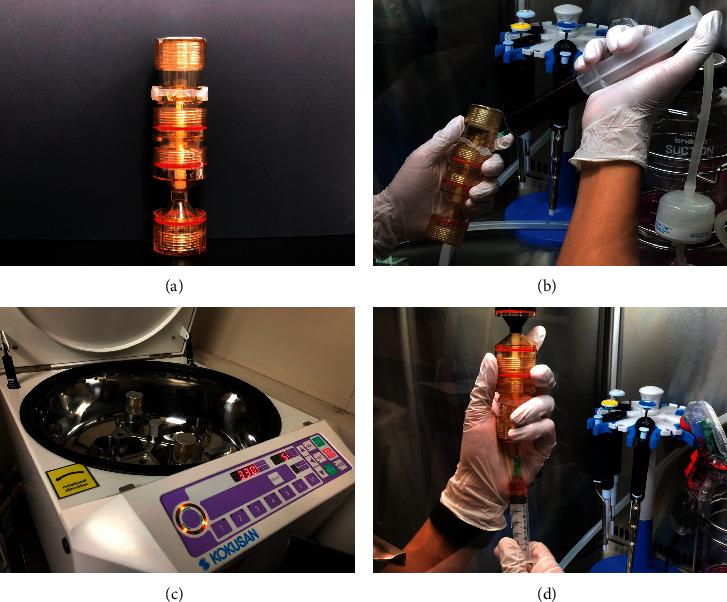
PRP preparation using the TriCell PRP Kit. (a) Collection tube from the TriCell PRP Kit before use. (b) Injection of whole blood into the upper part of the tube. (c) Centrifugation of the tube. (d) Collection of buffy coats containing platelets after adjusting platelet separation closing gap between the upper and lower parts.

A total of 3 mL ACD-A were mixed with 30 mL blood. The TriCell PRP Kit was used for PRP preparation (Figure 1). A total of 4 mL of PRP buffy coat were collected in a 5 mL syringe according to the manufacturer's instructions.

### 2.3. Counts of Blood Cells

Blood cells in whole blood and PRP extract were measured by using a blood cell counter (LC-661, FUKUDA Inc., Tokyo, Japan). Platelets, erythrocytes, and leukocytes were counted.

### 2.4. ELISA

PRP was preserved as follows: A: 1 h storage in room temperature (RT), B: 1 h storage at −20 °C, C: 24 h storage at 4°C, and D: 24 h storage at −20°C. Frozen PRP was thawed gradually in room temperature. The growth factors in each PRP that was extracted by double-spin methods and the TriCell PRP kit (D-PRP and T-PRP, respectively) were measured using ELISA kits (R&D Systems). PRP samples were assayed for PDGF-BB, PDGF-AB, EGF, and VEGF per the manufacturer's instructions.

### 2.5. Rabbit Model

Eighteen female Japanese white rabbits (weight, 1.8 to 2.3 kg; Kitayama Labes Co., Ltd., Ina, Nagano) were maintained in accordance with Institutional Animal Care and Use Committee guidelines and the Association for Research in Vision and Ophthalmology Statement for the Use of Laboratory Animals in Ophthalmic and Vision Research. Animal procedures were approved by the animal committee of Toho University (19-22-382). Rabbits were anesthetized by an intramuscular injection with 10 mg/kg xylazine hydrochloride (FUJIFILM Wako Pure Chemical Corp.) for all procedures. Rabbits were euthanized by an overdose of pentobarbital sodium. Right eyes of rabbit were marked with a 9 mm diameter trephine (Handaya, Tokyo, Japan), and the corneal epithelium within the marked area was removed with a sterile blade. The left eye of each rabbit was left untreated. Eye drop of PRP derived from human blood of one healthy volunteer (D-PRP or T-PRP) or phosphate buffer solution (PBS) as the control was instilled in rabbit cornea four times per day after epithelium removal. Rabbit eyes were evaluated at 6, 12, 24, 48, 72, 96, and 120 h by portable slit-lamp biomicroscopy (SL-17; KOWA, Tokyo, Japan), and images of the epithelial defect after fluorescein staining were captured. Photographs were analyzed using ImageJ software (http://rsb.info.nih.gov/ij). Outlines of each epithelial defect were manually traced, and area of the epithelial defect was measured using the application tool of the software. Wound healing ratio was measured as 100−epithelial defect area/epithelial defect area at 0 h × 100. Experiments were performed with a minimum of three animals per experimental group and repeated at least twice.

### 2.6. Histology

Both eyes of each rabbit were removed immediately after complete re-epithelization was observed. Globes were fixed in phosphate-buffered 10% formalin solution for 24 h. Eyes (*n* = 4 per group) were sectioned and stained with hematoxylin and eosin. Corneal epithelial damage was examined by light microscopy (DMi1; Leica, Wetzlar, Germany). Thickness of the corneal epithelium at the center, limbus, and middle of the center and limbus cornea were measured by ImageJ software.

### 2.7. Transmission Electron Microscopy (TEM)

The cornea was prepared via prefixation using 2% glutaraldehyde at 4°C, washing overnight at 4°C, postfixation using 2% osmium tetraoxide for 2 h at 4°C, dehydration, substitution using propylene oxide for 30 min and a mixture of propylene oxide and epoxy resin for 2 h, embedding, and sectioning. Ultrathin sections were mounted on 200-mesh copper grids and stained with 2% uranyl acetate for 15 min and then with lead solution for 5 min. Sections were observed with a Hitachi H-7600 electron microscope at 100 kV.

### 2.8. Statistical Analysis

The paired *t*-test was used to compare blood cells and growth factors, while the Tukey–Kramer method was used to compare wound healing ratio and corneal thickness. *P* ≤ 0.05 was considered statistically significant. All analyses were conducted using JMP version 11 statistical analysis software (SAS Institute, Inc., Cary, 150 NC, USA).

## 3. Results

### 3.1. Cell Counts in PRP

First, 30 ml of whole blood was collected from 4 healthy male volunteers. Thereafter, 4 ml of T-PRP and 14 ml of D-PRP were extracted. The experiments were repeated two or three times per volunteer. Prior to PRP extraction, bloods cells in D-PRP and T-PRP were compared to those in whole blood (Table 1). Platelet concentration of D-PRP and T-PRP was significantly higher than that of whole blood (*P* < 0.0001). In addition, platelet concentration of T-PRP was richer than that of D-PRP (*P* < 0.0001). Enrichment rate of D-PRP and T-PRP, which was calculated as platelets of PRP/platelets of whole blood, was 1.87 and 3.02, respectively. T-PRP, but not D-PRP, had a greater number of leucocytes and lymphocytes than whole blood (*P* < 0.05), while both D-PRP and T-PRP were not rich in erythrocytes.

### 3.2. Growth Factors

**Figure 2:**
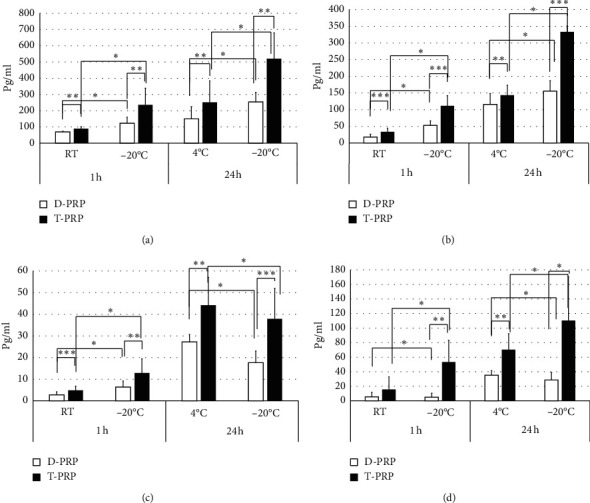
Growth factors in PRP: (a) PDGF-BB, (b) PDGF-AB, (c) EGF, and (d) VEGF. RT, room temperature; ^*∗*^*P* < 0.05; ^*∗∗*^*P* < 0.01; ^*∗∗∗*^*P* < 0.001 (*n* = 6–9).

We compared the growth factors among D-PRP and T-PRP at various conditions (Figure 2). As a result, we found that the concentration of all growth factors in the T-PRP stock conditions was higher than that in D-PRP. In addition, the concentration of all growth factors in PRP stocked at 24 h was higher than that stocked at 1 h; the concentration of PDGF-BB and PDGF-AB after freezing and thawing was higher than that stocked at RT or 4°C. T-PRP after a 24 h storage at −20°C had a markable concentration of PDGF-BB and PDGF-AB. Meanwhile, after 1 h, the concentration of EGF after freezing and thawing was higher while that after 24 h was lower than that stocked at RT or 4°C. Moreover, VEGF concentration after freezing and thawing was higher in T-PRP alone than in that stocked at RT or 4°C.

### 3.3. Efficacy of PRP in the Rabbit Eye

**Figure 3:**
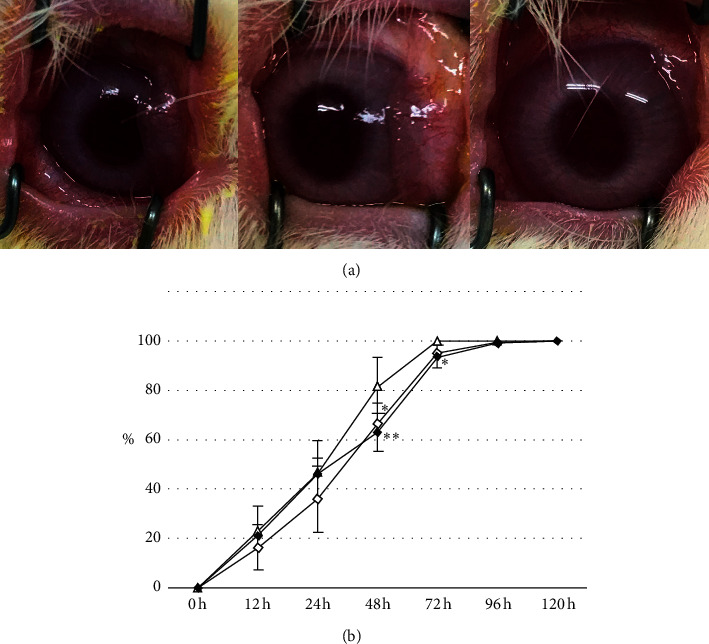
Corneal wound healing of eyes treated with D-PRP and T-PRP. (a) Representative clinical findings of the cornea after treatment with control (A), D-PRP (B), and T-PRP (C). (b) Comparison of wound healing rate between PRP treatment and control. Opened triangle, control; opened rhombuses, D-PRP; closed rhombuses, T-PRP. ^*∗*^*P* < 0.05 and ^*∗∗*^*P* < 0.01. Data represent mean ± standard deviation (SD) (*n* = 6).

The effect of the PRP eye drop after freezing and thawing, which may release higher growth factors, was investigated in the corneal epithelial defect model. At all observation points, the cornea treated with D-PRP, T-PRP, and control appeared clear, with no infiltration, and were neovascular (Figure 3(a)). The rate of wound healing in eyes treated with D-PRP and T-PRP was significantly slower than those treated with control (Figure 3(b)).

### 3.4. Histology

**Figure 4:**
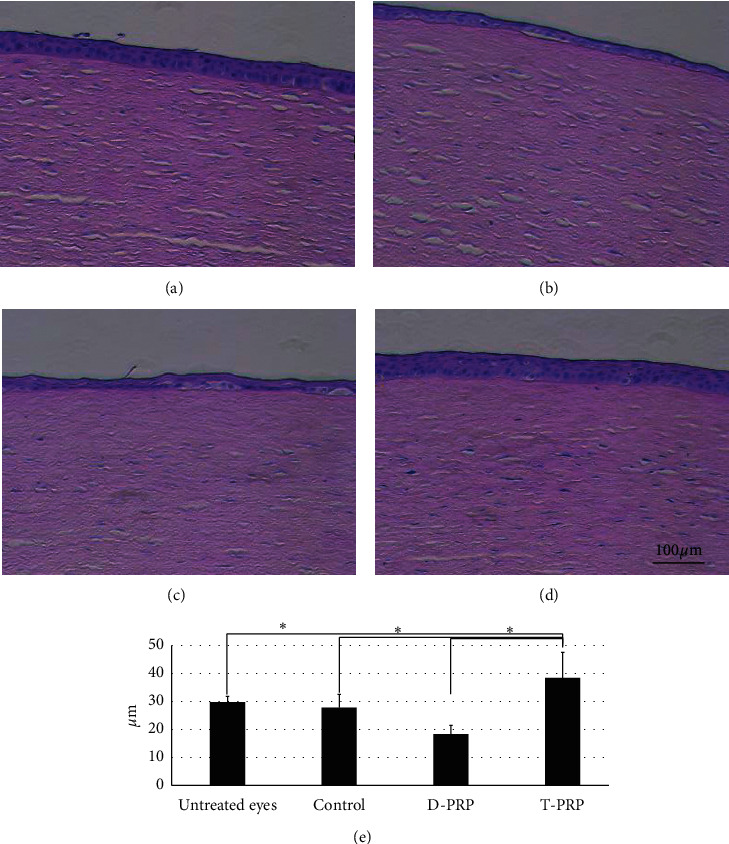
Histology of the cornea after complete wound healing. (a) Untreated eye, (b) eyes treated with control, (c) eyes treated with D-PRP, (d) eyes treated with T-PRP, and (e) thickness of the epithelial layer; ^*∗*^*P* < 0.05; *n* = 4.

Immediately after repairment of the epithelial defect, histology of the cornea was checked in the tested group (*n* = 4). As a result, no cells other than epithelial cells were found in the epithelium and stroma of all groups. Thereafter, the healed epithelium was analyzed in detail. Successful cell stratification (multilayer), not cell size or cell swelling, was observed in eyes treated with T-PRP only, similar to results obtained in untreated eyes. A space was found between the epithelium and basal membrane at the center of the cornea in 3 of 4 control eyes, 2 of 4 D-PRP-treated eyes, and 1 of 4 T-PRP-treated eyes (Figure 4(a)). Thickness of the epithelial layer between the basal membrane and superficial part of epithelial cells at the center of cornea was measured immediately after wound healing. Eyes treated with T-PRP (38.41 ± 9.10 *μ*m) were found to be significantly thicker than untreated eyes (29.74 ± 2.04 *μ*m) and eyes treated with control (27.77 ± 4.76 *μ*m) or D-PRP (18.32 ± 3.14 *μ*m) (*n* = 4, *P* < 0.05) (Figure 4(b)).

### 3.5. TEM

**Figure 5:**
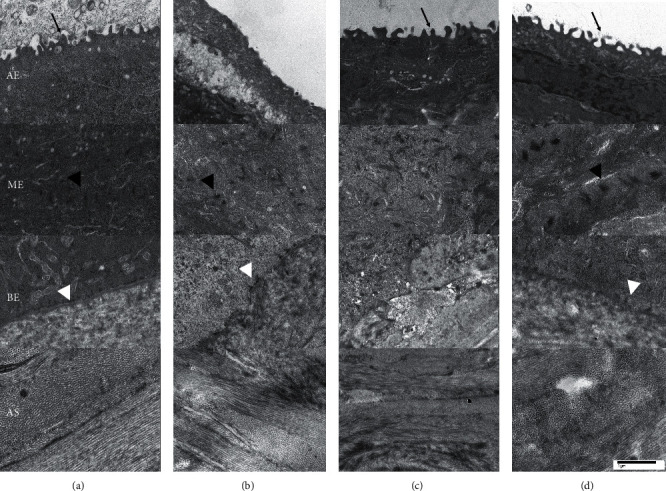
Representative TEM images. (a) Untreated eye, (b) eyes treated with control, (c) eyes treated with D-PRP, and (d) eyes treated with T-PRP. AE, atypical epithelium; ME, middle epithelium; BE, basal epithelium; AS, anterior stroma; black arrows, microvilli; black arrow heads, desmosome; white arrow heads, basal membrane.

Epithelial layers in two eyes of each treated group were observed by TEM (Figure 5). As a result, microvilli were identified on the apical side of the epithelium in eyes treated with D-PRP and T-PRP and untreated eyes. In addition, a desmosome was found between the epithelial cells in all tested eyes and a clear desmosome was observed in eyes treated with T-PRP. Untreated eyes and T-PRP-treated eyes showed similar morphology of basal epithelium. Anterior stroma was similar in all tested eyes.

## 4. Discussion

Many published papers have described the efficacy of the PRP eye drop for the treatment of ocular surface disease. Alio et al. demonstrated that the PRP eye drops are effective for the treatment of various corneal diseases such as corneal ulcer and dry eye [8–10, 12, 17–19]. By using the single-spin methods, these authors prepared PRP eye drops containing ∼340.07 ± 98.37 × 10^3^/*µ*l of platelets (platelet enrichment rate: 1.71) and few leucocytes [10]. Anitua et al. used plasma rich in growth factors (PRGF) synthesized via another platelet-rich preparation with the Endoret ophthalmology kit (BTI Biotechnology Institute, S.L., Miñano, Álava, Spain) that avoids the buffy coat which contains the leukocytes [24]. The PRP prepared using the TriCell PRP kit in this study may include higher or similar amounts of platelets relative to previous studies. The TriCell kit, which is divided into the trapping erythrocyte part and another part containing serum, was used to collect the buffy coat, including platelets and leucocytes, from the two parts. This kit may therefore concentrate platelets as well as leukocytes, especially lymphocytes. Leukocyte-rich PRP (Lr-PRP) can also be concentrated using the kit. Although the advantages and disadvantages of Lr-PRP have been constantly debated in fields other than ophthalmology, little is known about the role of leucocytes in PRP-activated corneal regeneration. Lr-PRP is often used to treat open traumatic injuries as it may have antimicrobial effects [25, 26]. In addition, leukocytes in PRP may stimulate the immune response against infection and promote chemotaxis, proliferation, and differentiation of cells [27]. On the other hand, leukocytes in PRP may release proinflammatory cytokines, such as interleukin-1*β* (IL-1*β*) and tumor necrosis factor-*α* (TNF-*α*), which produce destructive proteases and induce catabolism of the ECM [28, 29].

In this study, growth factors, especially PDGF, were greater in T-PRP than in D-PRP. As T-PRP had a greater number of platelets than D-PRP, T-PRP may produce growth factors. Platelet-leukocyte interaction could also increase the release of growth factors such as PDGF [30]. To release growth factors from platelets, platelets are either activated or destroyed. Calcium chloride addition to PRP is widely used for platelet activation. As platelet activators such as calcium and thrombin may be found on the ocular surface, platelet activation prior to eye instillation may not be necessary. Moreover, adding a drug to PRP may increase the cost and allow strict control. Because freeze/thawing is performed to physically damage platelet membranes and initiate the release of the granule content, freezing and thawing could increase the release of growth factors [31]. By performing freezing and thawing for 24 h in this study, the concentration of PDGF but not VEGF increased. A previous study revealed that cryopreservation of PRP is safe and induces the proliferation and production of ECM components in chondrocytes and synoviocytes [31]. For PRP eye drops, previous studies demonstrated that freezing and thawing PRP increased the growth factors and were effective for the treatment of ocular surface disease [14]. In the clinical setting, extracted PRP should be frozen prior to use as an eye drop.

We compared the efficacy of D-PRP to that of T-PRP for the de-epithelization model. As our in vitro data showed that T-PRP is rich in lymphocytes, a careful observation was performed to determine whether corneal inflammation appeared after instillation of the T-PRP eye drops. Slit-lamp observation and histology did not reveal any signs of inflammation such as corneal infiltration and haze. Thus, L-PRP could not induce inflammation in the corneal de-epithelization model. Because the pathological condition of corneal surface diseases such as corneal ulcer and PED may be related to inflammation, further investigations on the role of leukocytes in PRP eye drop are needed in the corneal inflammation model. However, as L-PRP has antimicrobial activity [25, 26], it may be effective when used to treat or prevent infections in the cornea.

Unexpectedly, compared to the control, both D-PRP and T-PRP caused a slower healing of the corneal epithelial defects. In a previous study using a rabbit model, PRGF was demonstrated to promote corneal re-epithelization. Although T-PRP and D-PRP may not display the function of cell migration promoters, instillation of eye drops for a total of 4 times may not be sufficient to promote wound healing. PRP storage condition and the effective period have not been investigated but are crucial as growth factors might be deactivated during long-term preservation. Human PRP and growth factors might not display strong efficacy for the regeneration of rabbit corneal epithelium. Nonetheless, relative to wound healing speed, histology demonstrated stratified epithelial cells in eyes treated with T-PRP. Moreover, TEM revealed normal junctions such as desmosome in T-PRP-treated eyes. Thus, T-PRP may promote to normalize epithelial cells. Topical PDGF application to the cornea increased fibroblasts at the wound junction and the production of type III and type IV collagen [32]. Previous studies suggested that PDGF may play an important role in corneal wound healing [33, 34]. Homeostasis of corneal epithelium is well known as the XYZ hypothesis (X, proliferation of basal epithelial cells; Y, centripetal movement of peripheral cells; and *Z*, epithelial cell loss from the surface) [35]. Since eyes treated with T-PRP showed thicker corneal epithelium, it may stimulate *X* rather than Y. We need further investigations about cell proliferation and evaluation of Ki67 staining using immunohistological chemistry in future. When the clinical application of T-PRP for ocular surface disease is considered, it could be very important to strengthen epithelial cells. Thus, T-PRP may be a good treatment option for corneal disease such as RCE when the epithelial barrier is weak or epithelial cell-basal membrane adhesion is poor. Moreover severe ocular surface disorders which have weaken corneal epithelium, such as Stevens–Johnson syndrome, ocular cicatricial pemphigoid, and severe thermal or chemical injury, may be susceptible to T-PRP.

In conclusion, when TriCell is used to prepare PRP, the resulting PRP is rich in both platelets and leukocytes and releases high amounts of growth factors after freezing and thawing. Although the efficacy and mechanism of leukocyte-rich PRP may be unclear, it may promote cell proliferation, but this may not be accompanied by strong corneal inflammation.

## Figures and Tables

**Table 1:** Comparison of the number of blood cells (/*μ*l).

	Whole blood	D-PRP *P* value vs. whole blood	T-PRP *P* value vs. whole blood	*P* value (D-PRP vs. T-PRP)
Red blood cell/*µ*l (mean ± SD)	478.14 ± 29.99 × 10^4^	1.36 ± 0.50 × 10^4^; *P* < 0.0001	11.18 ± 4.83 × 10^4^; *P* < 0.0001	*P* = 0.5325
Leucocyte/*µ*l (mean ± SD)	5.09 ± 1.09 × 10^3^	0.41 ± 0.24 × 10^3^; *P* < 0.0001	10.09 ± 4.29 × 10^3^; *P* < 0.0001	*P* < 0.0001
Lymphocyte/*µ*l (mean ± SD)	1.94 ± 0.40 × 10^3^	0.21 ± 0.30 × 10^3^; *P* = 0.0017	6.79 ± 2.41 × 10^3^; *P* < 0.0001	*P* < 0.0001
Neutrophil/*µ*l (mean ± SD)	2.89 ± 0.80 × 10^3^	0.02 ± 0.03 × 10^3^; *P* < 0.0001	2.62 ± 2.39 × 10^3^; *P* = 0.0854	*P* < 0.0001
Platelet/*µ*l (mean ± SD)	22.26 ± 3.46 × 10^4^	41.36 ± 8.43 × 10^4^; *P* < 0.0001	67.02 ± 13.55 × 10^4^; *P* < 0.0001	*P* < 0.0001
Enrichment rate		1.87	3.02	

SD, standard deviation; D-PRP, PRP extracted by double-spin methods; T-PRP, PRP extracted by the TriCell PRP Kit. Enrichment rate, platelets of PRP/platelets of whole blood; *n* = 10–12.

## Data Availability

The data used to support the findings of this study are included within the article.
